# Trends in Nutritional Status and Dietary Behavior in School-Aged Children with Congenital Heart Defects

**DOI:** 10.3390/children11101264

**Published:** 2024-10-19

**Authors:** Dominik Tobias, Paul Christian Helm, Ulrike Maria Margarethe Bauer, Claudia Niessner, Sigrid Hahn, Jannos Siaplaouras, Christian Apitz

**Affiliations:** 1 Division of Pediatric Cardiology, University Children Hospital, 89075 Ulm, Germany; dominik.tobias@uni-ulm.de; 2 National Register for Congenital Heart Defects, Competence Network for Congenital Heart Defects, 13353 Berlin, Germany; 3 Institute of Sports and Sports Science, Karlsruhe Institute of Technology, 76131 Karlsruhe, Germany; 4 Oecothrophology and Nutritional Science, University of Applied Sciences, 92224 Fulda, Germany; 5 Pediatrics and Interprofessional Care, University of Applied Sciences, 92224 Fulda, Germany

**Keywords:** congenital heart disease, pediatric cardiology, nutrition, fast food, obesity, KiGGS

## Abstract

**Background:** Malnutrition and poor weight gain has been reported in infants with congenital heart defects (CHDs); however data in older children with CHDs are limited. In order to obtain representative data on the nutritional status, dietary behavior, and potential influencing factors in school-aged children with CHDs, we performed a nationwide online survey. **Methods:** Patients aged 6 to 17 years registered in the German National Register for CHDs were asked to participate in this study by completing the German Health Interview and Examination Survey for Children and Adolescents (KiGGS) eating study questionnaire in order to assess their self-reported dietary habits. The use of the same questionnaire enabled a comparison with a representative subset of 4569 participants of the KiGGS study. **Results:** A total of 894 patients (mean age 12.5 ± 3.0 years; 47.2% female) were enrolled. Patients were allocated according to anatomic complexity into simple (23.8%), moderate (37.8%), and complex CHDs (38.4%). The consumption of sugar-containing food (*p* < 0.001) and fast food (*p* < 0.05) was significantly lower among the CHD patients than in the healthy children. Children with CHDs showed significantly lower body mass index (BMI) percentiles (*p* < 0.001) compared with their healthy peers, while children with complex and moderate CHDs had the lowest BMI. While in CHD patients, the BMI percentiles were not related to unhealthy food, there was a strong correlation with the CHD severity and number of previous interventions (*p* < 0.01). **Conclusions:** According to this nationwide survey, school-aged children with complex CHD are at risk of undernutrition, which is not due to dietary habits but to CHD severity and repeated surgery.

## 1. Introduction

A balanced and needs-based diet is important at every stage of life but plays a special role in childhood and adolescence. Adequate nutrition is crucial for the growth, development, and well-being of children and adolescents [1]. Consequently, it is especially important to assess their nutritional status and dietary habits in order to identify deficiencies and potential health issues [2]. Dietary behavior significantly influences one’s nutritional status and the risk of underweight or overweight [3]. Data from the German Health Interview and Examination Survey for Children and Adolescents (KiGGS) “Nutrition Study as a KiGGS Module” reflect the current situation regarding the nutrition of children and adolescents in Germany and showed that most adolescents do not eat enough fruit, vegetables, and plant-based foods. The consumption of meat, sausage products, sweets, soft drinks, and snacks is clearly too high. This nutritional behavior results in a relevant number of 6-to-17-year-olds in Germany who are overweight or obese [4]. While these results are worrying and show that this issue is relevant to public health, particularly with regard to the risk of cardiovascular disease in adulthood [5], it remains unclear whether this nutritional situation of the general population also applies to children and adolescents with chronic diseases, such as congenital heart defects (CHDs), the most frequent congenital malformation diagnosed in children [6].

It is known from previous reports that infants with CHDs, despite normal weights at birth, often face nutritional challenges in their early months, affecting growth and development, and resulting in malnutrition and underweight [7,8,9,10,11,12,13,14,15,16]. Malnutrition in children with CHDs is multifactorial, influenced by factors like increased caloric needs, difficulties in nutrition provision, fluid limitations, and feeding tolerance issues [13,15,16]. Children with CHDs often struggle with vomiting and may develop an oral food aversion [17]. Postoperative risks include interruptions in feeds, vocal cord dysfunction, and chylothorax. Malnutrition in these children leads to prolonged hospital stays, increased ventilation duration, more intensive care unit days, and higher complication rates [13,14,18,19,20,21,22].

Previous data in children with CHDs were predominantly focused on malnutrition in infants and young children [7,10,11,12,13,18]; however, data on nutritional status and dietary behavior in school-aged children and adolescents are limited so far.

Therefore, we conducted a nationwide survey in collaboration with the German National Register for Congenital Heart Defects (NRCHD) in order (I) to obtain representative data regarding the nutritional behavior in children and adolescents with CHDs living in Germany, (II) to detect differences compared with children/adolescents without CHDs using an appropriate reference cohort, and (III) to study factors potentially influencing the nutritional status in young CHD patients.

## 2. Methods

### 2.1. Study Design

This cross-sectional online survey “E-BAHn” (German acronym for “Ernährung Bei Angeborenen Herzfehlern”, English translation: “Nutrition in CHD patients”) was conducted from November to December 2021. Patients aged 6 to 17 years (on the date of launching the survey) were identified and contacted via the database of the NRCHD, the largest European register for CHD patients, and were asked to participate in the online survey. This study was conducted in accordance with the Declaration of Helsinki and approved by the Institutional Ethics Committee of Charité University Medicine Berlin (protocol code EA2/247/20, date of approval 9 December 2020).

### 2.2. KiGGS Reference Cohort

The KiGGS study is part of the national health monitoring system of the Robert Koch Institute (RKI). Within the KiGGS survey, a study with an in-depth focus on nutrition was previously performed, with the aim of providing representative data on food consumption and nutritional status among children and adolescents living in Germany [23]. By using these data from the KiGGS study, a healthy reference cohort sample of 4569 children and adolescents with comparable age and sex distributions was available.

### 2.3. Survey Instruments

The KiGGS eating study questionnaire included 94 questions: 60 questions regarding nutritional behavior and the frequency of consumption of particular food groups during the last 4 weeks, 19 questions regarding physical activity, and 15 general and anthropometric questions, including self-reported body weight and height.

### 2.4. Food Group Quantities

For the statistical analysis, the daily food amounts consumed were summarized in 4 different food groups: milk dishes, sugar-containing food, fast food, and fruits and vegetables (Table 1). Table 2 shows the answer options in the questionnaire regarding consumption frequency and the corresponding categories for statistical analysis. To obtain results for the evaluation of the entire food groups, the newly generated variables (consumption frequency variables) of the respective group were summed up to a new variable. This variable formed the total score of the corresponding food group. This total score was then divided by the number of consumption frequency variables contained in the group, which resulted in a final score variable (consumption frequency score) for each food group. This had a range of values from 1 to 4, similar to a scale, with a higher score indicating increased or more frequent consumption. In order to keep the variables comparable, the value range was rounded off pursuant to common rules, resulting in the food group consumption frequency scores 1–4 (Table 3).

### 2.5. BMI Analysis

In order to allow for a comparison of the BMI data for all age groups, BMI percentiles were used and were categorized as severely underweight, underweight, normal weight, overweight, and obese according to the definitions of Kromeyer-Hauschild et al. [24,25] (Table 4).

### 2.6. Statistical Analysis

Values of continuous variables are reported as the mean ± standard deviation. Pearson’s chi-square test was used for group comparisons, including nominal data; *t*-test for metrics; and the Mann–Whitney-U-test for ordinal variables. In order to assess the impact of potential contributing factors on nutritional behavior, Spearman correlation analysis was used. IBM SPSS statistics version 28.0 (IBM Inc., Armonk, NY, USA) was used for the statistical analyses. A significance level of *p* ≤ 0.05 was applied.

## 3. Results

### 3.1. Patient Characteristics

A total of 1647 patients agreed to participate in the online E-BAHn survey, and 894 patients (mean age of 12.5 ± 3.0 years; 47.2% female) completed the survey and were available for evaluation. The study participants were allocated according to the anatomic complexity (Warnes classification) [26] into simple (23.8%), moderate (37.8%), and complex CHDs (38.4%).

In total, 30.9% of the included patients had no available information on surgical/interventional treatment or untreated CHD, 46.5% had received 1–3 operations/interventions, and 22.6% even more than 3 operations/interventions.

In 106 patients (11.9%), a genetic syndrome and/or chromosomal disorder was present, most commonly Down syndrome (45 patients, 5%) and Di-George syndrome (12 patients, 1.3%).

### 3.2. Food Consumption

The majority of children with CHDs and participants of the KiGGS study indicated a frequent consumption of milk dishes (57.6% vs. 63.3%), while the consumption was significantly higher in the KiGGS cohort (*p* < 0.001). Almost 50% of CHD patients indicated only rare consumption of sugar-containing food, while 60.4% of the healthy peer group indicated frequent consumption (*p* < 0.001). The majority of CHD patients and KiGGS participants (87.2% vs. 88.4%) did consume fast food only rarely; however, there was still a significant difference between both groups, with a higher consumption among the healthy group (*p* < 0.05) (Table 5). Remarkably, the consumption of fruits and vegetables was similarly distributed in both groups (*p* = 0.587), approximately every second participant indicated frequent consumption.

### 3.3. Nutritional Status

The percentile-defined BMI categories were largely normally distributed among the CHD patients. More than 71% of the subjects presented with a normal weight. Remarkably, overweight (6.5%) and obesity (3.6%) were more rarely detected than in the KiGGS cohort (8.9% and 6.8%, respectively).

**Figure 1:**
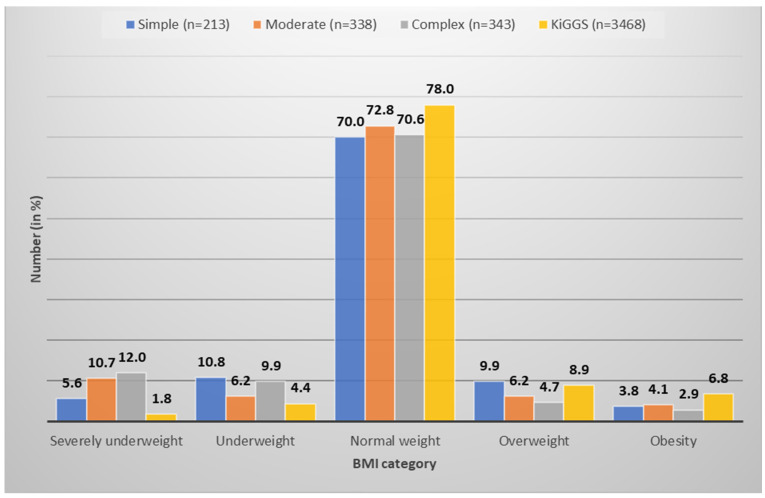
Distribution of body mass index (BMI) percentile-defined categories based on congenital heart defect (CHD) severity and healthy patients (KiGGS). Overweight and obesity were more rarely detected in CHD patients. More common was underweight, especially with complex and moderate CHDs.

In contrast, remarkably more CHD patients were underweight (8.7%) and severely underweight (10.0%) compared with the KiGGS cohort (4.4% and 1.8%, respectively). Even 12% of those with complex CHD presented with severe underweight (Figure 1).

**Figure 2:**
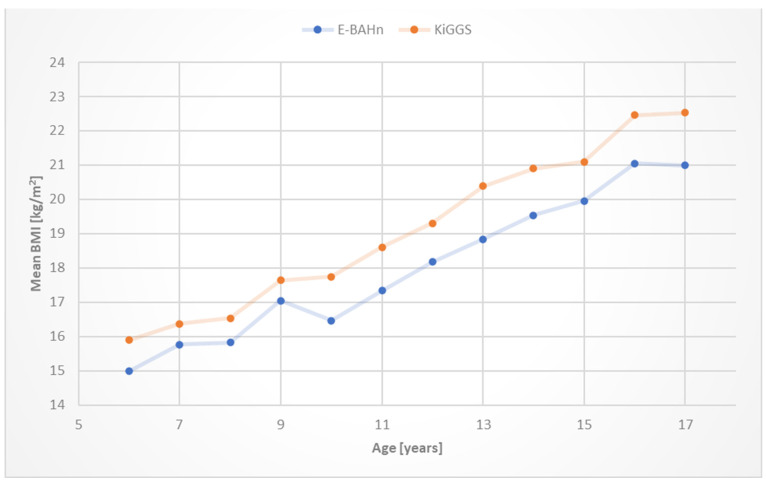
Age-related mean body mass index (BMI) of children with congenital heart defects (CHDs) (E-BAHn; n = 894) and healthy reference group (KiGGS; n = 3468). Children with CHDs showed significantly lower BMI (*p* < 0.001).

**Figure 3:**
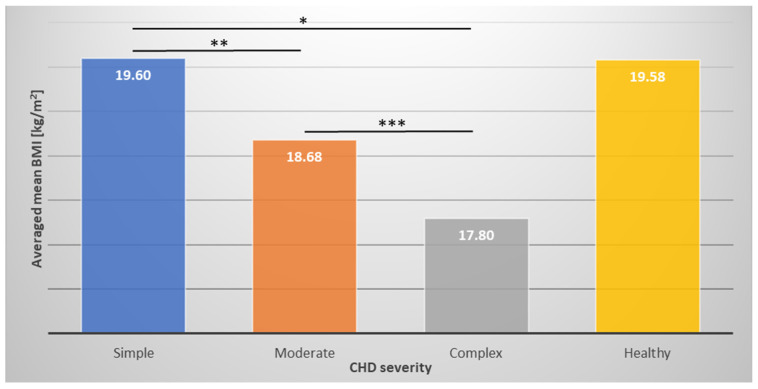
Averaged mean body mass index (BMI) in relation to congenital heart defect (CHD) severity compared with the healthy reference group. Children with complex CHDs had the lowest BMI. * *p* < 0.001; ** *p* = 0.005; *** *p* = 0.002.

Consequently, compared with their healthy peers, children with CHDs showed a significantly lower body mass index (BMI) (*p* < 0.001), while the difference in BMI appeared most pronounced in older children (Figure 2). When we compared the averaged mean BMI between the CHD severity classes, there was no difference between simple CHD and the healthy reference group; however, there was a significant difference between simple and moderate (*p* = 0.005), simple and complex (*p* < 0.001), and moderate and complex CHDs (*p* = 0.002), while patients with complex CHDs had the lowest BMI (Figure 3).

### 3.4. Factors That Influenced Nutritional Status

Correlation analyses demonstrated a significant impact of physical activity (*p* = 0.007) and consumption of sugar-containing food (*p* < 0.001) on the BMI percentiles in the KiGGS cohort (Table 6). In contrast, there was no correlation found between the BMI percentiles and eating habits, as well as physical activity, among the children with CHDs. However, strong correlations with the BMI percentiles were found in this cohort for the CHD severity (*p* = 0.007) and number of previous interventions (*p* = 0.009).

## 4. Discussion

To the best of our knowledge, the present study was the largest cohort of school-aged children with CHDs that has been investigated for their nutritional status and dietary behaviour so far.

According to this nationwide survey, school-aged children with CHDs living in Germany showed significantly lower BMI than their healthy peers. Furthermore, children with complex CHDs were at risk of undernutrition, and this did not appear to be due to dietary habits but to CHD severity and repeated surgery.

Patients with CHDs have an elevated risk for acquired cardiovascular disease in adulthood, such as metabolic disease, stroke, and coronary artery disease, compared with healthy individuals [27,28]. Risk factors in children with CHDs include sedentary behavior, dyslipidemia, and subclinical atherosclerosis [29,30]. It is generally recognized that the development of atherosclerotic and metabolic disease manifesting in adulthood usually starts already in early childhood. This highlights the need for primary prevention; hence, recent guidelines for the management of CHD patients include lifestyle interventions and eating habit recommendations [31]. Thus, there is emerging consensus that physicians and healthcare professionals should encourage children and adolescents with CHD not only to adopt a physically active lifestyle but also to eat healthy diets [32,33].

Whether our results reflect that this already existing holistic approach to the care of children with CHDs might have resulted in a more health-conscious lifestyle can only be speculated. Nevertheless, our results are remarkable and were able to demonstrate that school-aged children with CHDs ate unhealthy diets less frequently, especially sugar-containing food or fast food, compared with healthy children of the same age in Germany.

Another potential reason for these results might be the fact that the topic of healthy eating has been addressed in many social media platforms in recent years, i.e., Facebook or Instagram, particularly in connection with beauty role models. This could also have contributed to a change in the dietary behavior of children and adolescents, especially in children with more screen time, for example, children with CHDs with a more sedentary behavior [29].

Remarkably, there was also a lower prevalence of obesity in children and adolescents with CHDs, which may have also been a result of healthier nutritional behavior. The increasing numbers of children with obesity in the general population was demonstrated in the KiGGS study and might be, at least in part, a consequence of the consumption of unhealthy diets, particularly fast food and sugar-containing food [34]. Recently, the link between unhealthy dietary patterns and the risk of adiposity was also demonstrated in children with CHDs [35].

More frequently seen in our patient cohort was underweight, especially in patients with complex CHDs. Our results regarding potential reasons for underweight included an important impact of CHD severity and the number of interventions, which was most frequently the case in patients with complex CHDs. Malnutrition, defined as an imbalance between nutrient requirement and intake, resulting in deficits of energy, protein, or micronutrients [36], might have a relevant impact on morbidity and mortality in patients with heart disease. Data from a large-scaled meta-analysis in adults with heart failure revealed malnutrition more than doubled the risk for all-cause mortality [37]. Several dietary interventions resulted in a significant reduction in mortality and readmission for heart failure. Malnutrition in children with CHDs also has significant implications for their health and well-being, including impaired growth and development, increased metabolic risk, cognitive and neurodevelopmental impairments, and immune function dysregulation. Malnutrition can also increase surgical risk (increased hospital length of stay, increased postoperative infection rates) [38].

Therefore, in patients with CHDs and undernutrition or overnutrition, timely proactive nutritional interventions should be considered and may help to prevent malnutrition with all its serious consequences. However, measures to promote positive changes in nutritional behavior should not only be addressed to children and young people and their parents but should also make it easier for them to make healthier and informed decisions in their everyday lives.

### Study Limitations

In this study, we used anthropometric measurements (BMI) in combination with a food frequency questionnaire for the assessment of nutritional behavior. Clearly, assessing nutritional behavior by multiple methods (including 24 h recalls, biomarkers, and body composition analysis) could be more precise; however, this might be difficult to apply in a large patient population. The strength of our presented study was the considerably large sample size and the inclusion of school-aged children, particularly with a relatively wide age range, even until adolescence. Additionally advantageous was the application of the same questionnaire as used by the KiGGS study, which allowed us to compare the obtained results with a Germany-wide representative reference cohort.

However, there were also some limitations of the used questionnaire: the obtained data were based on proxy or self-reports and might therefore be prone to bias, including recall bias and social desirability; if underweight, weight may be overestimated, while if overweight, weight may be underestimated.

The retrospective survey instrument recorded the usual medium-term diet over a reference period of four weeks. This method also required certain cognitive skills on the part of the respondents, such as remembering the foods eaten and portion sizes, as well as basic knowledge of food identification.

Due to the study design, the study results could only provide descriptive information and observe associations rather than figure out causal relationships between the nutritional status and potential contributing factors.

Since nutritional behavior might be affected by the life situation of the patients, as well as the organization of the healthcare and educational system, the results of this nation-wide survey may not be applicable to CHD patients outside of Germany. However, additional factors that may contribute to nutritional behavior, for example, socioeconomic status, cultural background, type of school, and screen time, were not evaluated.

In addition, perinatal data were not included in this study, as they were not consistently collected by the NRCHD. Neonates with CHDs may present with a lower birthweight and smaller head circumference [39,40]. In order to demonstrate that diet influences the growth of CHD patients vs. healthy peers with divergent effect, the delta weight or BMI between cases and controls should ideally be analyzed before there is a significant effect of diet (i.e., in the perinatal period). However, these perinatal data might be more relevant for the evaluation of nutritional status in small infants. Our study was primarily focused on school-aged children and adolescents (aged between 6 and 17 years), where the direct influence of the perinatal period might be lower.

## 5. Conclusions

According to this nationwide survey, school-aged children and adolescents with CHDs, especially those with hemodynamically significant disease, may be at risk of undernutrition. The awareness of this relevant issue can help to establish structures (i.e., thorough nutritional assessment and appropriate nutritional support) in order to gather more evidence on this topic, thus allowing for future studies that may address the impact of proactive nutritional interventions for the prevention of malnutrition on long-term outcomes and quality of life.

## Figures and Tables

**Table 1:** Foods summarized in 4 different food groups. Quantities were calculated on the basis of the amount of consumed food.

Milk Dishes	Sugar-Containing Food	Fast Food	Fruits and Vegetables
○ Milk ○ Cream cheese ○ Cheese ○ Curd cheese, yogurt, soured milk	○ Honey, jam ○ Nut nougat cream ○ Cakes, pies, sweet pastries ○ Chocolate/candy bars ○ Sweets (candy, fruit gum) ○ Ice cream ○ Biscuits ○ Lemonade with sugar	○ Burger, pita kebab ○ Sausage, meat ○ French fries ○ Pizza	○ Fresh fruit ○ Cooked fruit ○ Raw vegetables ○ Cooked vegetables ○ Legumes

**Table 2:** Answer options in the questionnaire regarding the consumption frequency and the corresponding categories for the statistical analysis.

Answer Options in the Questionnaire	Category (Consumption Frequency)
Never	Never (=1)
1× a month	Rarely (=2)
2–3× a month	
1–2× per week	Frequently (=3)
3–4× per week	
5–6× per week	
1× a day	Daily (=4)
2× a day	
3× a day	
4–5× a day	
>5× a day	

**Table 3:** Range of values and food group consumption frequency scores.

Range of Values	Rounded Food Group Consumption Frequency Score
1–1.49	1
1.5–2.49	2
2.5–3.49	3
3.5–4	4

**Table 4:** Percentile ranges of BMI categories.

BMI Category	Percentile Range
Severe underweight	<P3
Underweight	P3–<P10
Normalweight	P10–P90
Overweight	>P90–P97
Obesity	>P97

**Table 5:** Food consumption frequency of the different food groups in children with CHDs (E-BAHn) compared with healthy children (KiGGS).

Milk dishes (consumption score)	E-BAHn (%)	KiGGS (%)	Significance *p*
1	2.6	1.3	
2	32.9	27.0	*p* < 0.001
3	57.6	63.3	
4	6.9	8.4	
Sugar-containing food (consumption score)	E-BAHn (%)	KiGGS (%)	Significance *p*
1	1.5	0.6	
2	49.9	38.2	*p* < 0.001
3	48.0	60.4	
4	0.7	0.8	
Fast food (consumption score)	E-BAHn (%)	KiGGS (%)	Significance *p*
1	6.8	5.0	
2	87.2	88.4	*p* < 0.05
3	5.8	6.6	
4	0.1	0.0	
Fruits and vegetables (consumption score)	E-BAHn (%)	KiGGS (%)	Significance *p*
1	3.8	2.8	
2	44.2	47.0	*p* = 0.587
3	51.7	49.5	
4	0.3	0.8	

**Table 6:** Potential factors that influenced the BMI in patients with CHD (E-BAHn) and healthy children in Germany (KiGGS). Abbreviations: BMI = Body mass index; CHD = Congenital Heart Defect; n/a = not applicable.

BMI Dependent on:	E-BAHn Correlation Coefficient r	E-BAHn Significance *p*	KiGGS Correlation Coefficient r	KiGGS Significance *p*
Physical activity	0.027	0.428	−0.046	0.007
Fast food	0.036	0.284	−0.003	0.877
Sugar-containing food	0.007	0.827	−0.071	<0.001
CHD severity	0.090	0.007	n/a	n/a
Number of interventions	−0.087	0.009	n/a	n/a

## Data Availability

The original contributions presented in the study are included in the article, further inquiries can be directed to the corresponding author.
